# Exploring pain interference and self-perceived health status in children with osteogenesis imperfecta - a cross-sectional study

**DOI:** 10.1186/s12891-022-05825-5

**Published:** 2022-09-21

**Authors:** Anna Hallin Provenzano, Eva Åström, Kristina Löwing

**Affiliations:** 1 Department of Women’s and Children’s Health, Neuropediatric Unit, Karolinska Institutet, Karolinska vägen 37A7tr, 171 76 Stockholm, Sweden; 2 Department of Women’s and Children’s Health, Karolinska Institutet, Stockholm, Sweden; 3 Astrid Lindgren Children’s Hospital, Karolinska University Hospital, Karolinska Institutet, Karolinska vägen 37A7tr, 171 76 Stockholm, Sweden

**Keywords:** Children, Osteogenesis imperfecta, Pain, Quality of life, Self-perceived health status

## Abstract

**Background:**

Chronic pain may affect and interfere in children’s everyday life and can be present in children with Osteogenesis Imperfecta (OI). However, the knowledge is still sparse to what extent pain is present, how pain interfere in children’s everyday life and affect their self-perceived health status. The purpose of the study was therefore to explore presence of chronic pain, pain interference in daily life, and self-perceived health status in children with OI.

**Methods:**

Children with OI, aged 6–18 years, were recruited consecutively to this cross-sectional study. Participants answered a standardised interview including five pre-structured questions, and the Numeric Pain Rating Scale (NPRS), the Pain Interference Index, and a questionnaire concerning self-perceived health status the Patient Reported Outcomes Measurement Information System Pediatric-25 Profile v1.1 (PROMIS-25).

**Results:**

Twenty-eight children (median: 11 years, IQR 6) with OI type I, III, or IV participated. Pain was present in 27 of 28 children and interfered in their everyday life regardless of OI-type, sex, and age. The median NPRS for average pain intensity was 4 (IQR 2), the median for pain frequency was 2–3 times/week, and the median frequency of school absence due to pain was 2–3 times per month. The most common pain locations were back and feet. Pain in the feet was more frequently reported in children with type I (*p =* 0.032), and pain in the hip was more often reported in children ≥13 years (*p =* 0.011). The children were asked what they thought to be the cause of pain and the most frequent response was “walking long distances”. Self-perceived health status for mobility was lower than the general population, and lowest for children with type III (*p =* 0.016). Pain interference was associated with children’s self-perceived health status (r_s_ = 0.84, *p <* 0.001).

**Conclusion:**

Almost all children experienced pain, which interfered in children’s everyday lives, affected participation in various activities and was associated with reduced self-perceived health status. If children avoid physical activities because of pain, it might cause a vicious circle of inactivity, which further decreases bone density and increase the risk of fractures. The results emphasize the importance to offer adequate pain reducing interventions.

## Background

Osteogenesis Imperfecta (OI) is a rare genetic disorder characterised by osteopenia and bone fractures, caused by mutations in collagen type I in up to 90% of affected individuals [1]. Common symptoms are skeletal deformities, multiple fractures, ligament laxity, muscle weakness, short stature, skeletal pain, dental and hearing problems, and sometimes cardiac and respiratory symptoms. The Sillence classification that has been used since 1979 identifies four types of OI based on clinical and radiographic criteria, combined with pattern of inheritance [2]. OI-type I is the mildest and most common form, with an absence of major bone deformities; type II is perinatally lethal; type III is severe, with short stature, severe limb and spine deformities, reduced muscle strength and multiple fractures; type IV is variable in severity, but often intermediate between type I and III [2]. This classification system has successively expanded and presently, the genome data base Online Mendelian Inheritance in Man (OMIM) includes 22 types of OI (Type I-XXII). There is still no cure for OI but treatment with intravenous bisphosphonate has been beneficial [3]. Bisphosphonates have been shown to have several positive effects, and one of them is to alleviate pain [4]. In addition, children may receive orthopaedic surgery with rodding, and physiotherapy aiming to support motor development and physical activity, increase opportunities for participation in everyday life, improve muscle strength, reduce fractures and chronic pain [5].

Chronic pain has been reported to affect between 11 and 38% of children worldwide [6]. According to The World Health Organization (WHO), pain in children is a public health concern, causing unnecessary suffering, and is a multidimensional phenomenon with sensory, physiological, cognitive, affective, behavioural, and spiritual components [7]. In the most recent definition, the International Association for the Study of Pain defines pain as, “An unpleasant sensory and emotional experience associated with, or resembling that associated with, actual or potential tissue damage” [8]. Acute pain is classified as short-term pain, persisting less than 3 months, whereas chronic pain persists for longer than 3 months. Chronic pain is reported to be more frequent in girls and the prevalence increases with age [9].

Untreated pain may affect quality of life (QoL) negatively [7, 10]. WHO has defined QoL as “an individual’s perception of their position in life in the context of the culture and value systems in which they live, and in relation to their goals, expectations, standards and concerns” [7]. QoL is a broad concept including physical health, psychological state, personal beliefs, and social relationships. Therefore, pain might negatively impact on all aspects of activities in children’s everyday lives [10, 11]. However, QoL and health-related quality of life (HRQoL) are concepts that have been debated and disputed, and the term “self-perceived health status” has sometimes been suggested as an alternative [12]. Thus, in relation to the present study, the term “self-perceived health status” will be used hereinafter.

In children with Osteogenesis Imperfecta (OI), both acute and chronic pain have been reported [13]. However, the most commonly reported pain in OI is chronic pain [13]. Since chronic pain can affect and interfere in everyday life, pain assessment, including pain interference, is warranted [10]. Although, it is difficult to investigate pain in children with OI. Firstly, many children have adjusted their activities to avoid painful situations, and secondly many children have since an early age got used to pain and do not report it [14]. Thirdly, there is a lack of multidimensional pain assessment for children with OI [13]. To clearly understand how pain affects the individual’s life, it has been suggested that a multidimensional pain assessment should include three main dimensions of pain experience: a sensory-descriptive (place, intensity, duration, quality), a motivational-affective and a cognitive-evaluative dimension [15]. Even though access to one optimal assessment tool is lacking, it is important to use a combination of available assessments to understand and treat the pain. The overall aims of this study were therefore to explore presence of chronic pain, pain interference in everyday life, and self-perceived health status in children with OI.

## Methods

### Design

The study design was cross-sectional.

### Setting

University hospital with nationwide admission area.

### Participants

A consecutive recruitment of children with OI from six to 18 years old, with a regular visit to the OI-team at the hospital’s and with a referral to the physiotherapy department at Astrid Lindgren Children’s Hospital in Stockholm, Sweden. The inclusion of children started in February 2018 and was ended in October 2019. In case of a recent fracture the OI-team visit was postponed until the child has recovered, to receive a representative evaluation of the child’s habitual status**.** Exclusion criteria was children that did not understand the Swedish language (children who recently immigrated to Sweden). The children and their parents were informed about the study at their scheduled appointment at the hospital. If they agreed to participate, informed consent forms were provided and signed. The study was approved by the Regional Ethical Review Board in Stockholm (Dnr 2017/1136–31/2) and conducted according to the ethical guidelines of the Declaration of Helsinki.

#### Procedure

Data collection consisted of an onsite visit with an interview, which was completed by the child and the same researcher (KL) and took around 30–50 minutes. In addition, information concerning the child’s type of OI, occurrence of treatment with bisphosphonate (pamidronate) and the classification of mobility were captured from the medical record. Mobility was classified by the Wilson Mobility scale, an ordinal scale running from 1 (Functional walking without aid in all surroundings) to 9 (Sitting with support and no mobility) [16].

### Assessments

#### A pre-structured interview

A pre-structured interview with five questions was used. The child was asked about: 1. Absence or presence of pain, 2. Number of days with pain (daily, 2–3 days per week, once a week, 2–3 times per month, or never), 3. Absence from school as number of days that the child stayed at home or went home early due to presence of pain (daily, 2–3 days per week, once a week, 2–3 times per month, or never), 4. Pain location/locations (head, neck, arm, hand, back, hip, knee, feet, and other), and 5. What the child believed to be the cause of the pain (open question). An additional question addressed to the parents concerned the child’s total number of long bone fractures (the majority of the fractures were treated at the local hospital near the place in Sweden where the child was registered, and therefore not recorded in the medical records at the University hospital).

#### Numeric pain rating scale

Pain intensity was reported with the Numeric Pain Rating Scale (NPRS), a segmented numeric version scale (0–10) where “0” represents “no pain” and “10” is the worst pain imaginable. The child was also asked to rate the intensity when the pain was at its lowest, average, and at its highest. NPRS is considered to be a well-established instrument in paediatric populations and for children ≥6 years of age with chronic pain [17].

#### Patient reported outcome measurement information system Pediatric-25 profile v1.1

The Patient Reported Outcome Measurement Information System Pediatric-25 Profile v1.1.

(PROMIS-25) was used to assess self-perceived health status. PROMIS-25 is a multidimensional instrument for children that evaluates self-perceived health status during the preceding 7 days [18]. The paediatric version 1.1 is composed of seven dimensions: mobility, anxiety, depressive symptoms, fatigue, peer relationships, pain interference, and intensity. The questions within the domain, pain interference, ask about pain interference during walking, running, concentration, and sleep (other scale and questions than within PII, except sleep). The instrument consists of a self-rating scale from 0 (never) to 4 (always) for all domains except pain intensity, in which the scale ranges from 0 (no pain) to 10 (worst pain). All domains, except pain intensity, are composed of four questions generating a total raw score within each domain, which can be summarised into a PROMIS-25 total raw score. The total raw score within each domain can then be converted to separate T-scores and may be used to compare results with the general population [18]. The standardized T-score for the general population has a mean of 50 and a standard deviation of 10. A high T-score (≥60 points) for anxiety, depressive symptoms, fatigue, and pain interference shows a poorer outcome, as compared with the general population. A low score (≤40 points) on mobility and peer relationships indicates a poorer outcome as compared with the general population. PROMIS-25 has been tested in children with chronic pain and showed support for validity and responsiveness [19].

#### Pain interference index

The Pain Interference Index (PII) was used to comprehensively evaluate whether pain interfered with the child’s functioning in everyday life (schoolwork, friends, physical activity, mood, mobility, and sleep), as PROMIS-25 more briefly addresses these issues. PII has another scale and there are other questions than within PROMIS-25 except sleep. PII was created to capture the interference of pain in children with longstanding pain syndrome and is a multidimensional instrument containing six statements where the child rates how well a statement describes his or her condition during the past 2 weeks, by using a numerical scale, ranging from 0 (not at all) to 6 (very much) [20]. The psychometric properties of PII have been evaluated and the results have shown that the instrument is suitable for children with chronic pain [20].

### Statistical analysis

The SPSS version 26 was used for analysis. Nonparametric statistics were used. Descriptive data are presented as median, interquartile range (IQR), numbers and percentage. To compare groups, Mann-Whitney U test or Kruskal-Wallis were used accordingly. Age was categorised into three groups: ≤9 years, 10–13 and ≥ 13 years. The total raw score within PROMIS-25 was presented, and raw scores for the separate domains were converted into T-scores. The significance level was set at *p <* 0.05. Spearman’s correlation coefficient (r_s_) was calculated between the PII total score and the PROMIS-25 total raw score, and between the separate domains within PII and the T-cores within separate domains in PROMIS-25. The correlation was considered significant when *p <* 0.05 and r_s_ > 0.37 [21]. The following interpretation was used: r_s_: 0.00–0.37 negligible correlation, 0.37–0.50 low correlation, 0.50–0.70 moderate correlation, 0.70–0.90 high correlation, and 0.90–1.00 very high correlation [22].

## Results

In total 28 children, 17 boys and 11 girls, with a median age of 11 years (IQR: 6), participated in the study (table 1). Due to time limitations, one child declined to participate. The children came from all over Sweden and had OI-type I, III or IV. In total 17 out of 28 (61%) children received intravenous Pamidronate, initially monthly infusions were given and according to treatment response (DXA, vertebral growth) the intervals between treatments were prolonged. The mobility was classified according to Wilson’s Mobility scale (table 1).

**Table 1:** Descriptive statistics of the participants

Participants	
Age, mdn (IQR)	11 (6)
Sex, (*n =* 28) (%)	
Male	17 (61)
Female	11 (39)
**Use of Bisphosphonate**, (*n =* 28) (%)	
Yes	17 (61)
No	11 (39)
**Type of OI**, (*n =* 28) (%)	
Type I	18 (64)
Type III	7 (25)
Type IV	3 (11)
⁕**Fracture rate**, mdn (IQR)	8 (13)
**Wilson mobility scale**, (*n =* 28) (%)	
1 Functional walking without aid in all surroundings	15 (54)
2 Functional walking without aid in secluded surroundings	11 (39)
3 Functional walking with crutches in all surroundings	0
4 Walking with crutches in secluded surroundings	0
5 Functional walking with key walker in all surroundings	0
6 Walking with key walker in secluded surroundings	0
7 Reciprocal crawling with arms and legs	0
8 Any other form of locomotion	1 (3.5)
9 Sitting with support and no mobility	1 (3.5)
**Presence of pain**, (*n =* 28) (%)	
Yes	27 (96)
No	1 (4)
**Pain frequency**, (*n =* 28) (%)	
Daily	11 (41)
2–3 days / week	5 (19)
Once a week	2 (7)
Less than 3 times / month	9 (33)
Never	1
**Absence from school due to pain,** (*n =* 27) (%)	
Daily	0
2–3 days / week	4 (14)
Once a week	1 (4)
Less than 3 times /month	15 (56)
Never	7 (26)

Data is presented as number (n), percentage (%), median (mdn) and interquartile range (IQR). Mobility is classified according to Wilson mobility scale, a nine-level scale (1–9). ⁕ Parents indicated the child’s total number of long bone fractures

Pain was present in 27/28 (96%) of the participants. The median pain frequency was 2–3 times per week, and the median frequency of school absence due to pain was 2–3 times per month (table 1). The most frequently reported locations of pain were the back (16/27) and feet (15/27), followed by the arm (5/27), head (4/27), hip (4/27), knee (4/27), neck (1/27) and hand (1/27). Pain in the feet was more frequently reported in children with type I (*p =* 0.032), and pain in the hip was more often reported in children ≥13 years (*p =* 0.011). Nine children reported pain in one location and 18 reported pains in multiple locations (≥2). There was no significant difference regarding presence of multiple pain between OI-types, sex, age groups or presence of bisphosphonate treatment. The children were also asked about what they thought to be the cause of the pain (*n* = 24). The most frequent responses were “walking long distances” (7/24), followed by “any time” (4/24), “during activity” (2/24), and “all the time” (2/24). Many children additionally explained that remaining in prolonged sedentary positions caused pain. Pain intensity (NPRS) was reported by 27 children, without significant differences between OI-types, sex, or age groups (table 2). The summarised median value for PII (*n* = 27) was 11 (IQR 16), without significant differences between OI-types, sex, or age groups (table 2).

**Table 2:** Descriptive statistics for the Numeric Pain Ratings Scale (NPRS), the Pain Interference Index (PII) and the Patient Reported Outcome Measurement Information System Pediatric-25 Profile v1.1 (PROMIS-25)

Assessment	Median (IQR) All children	Median (IQR) Type I	Median (IQR) Type III	Median Type IV
**NPRS** ^**1**^ **(*n =*** **27)**				
NPRS average	4 (2)	4 (2)	4 (2)	5
NRS highest	8 (4)	8 (4)	8 (4)	10
NPRS lowest	1 (2)	1 (2)	1 (3)	1
**PII** ^**2**^ **(*n =*** **27)**				
Schoolwork	2 (3)	1 (3)	3 (3)	3
Leisure activity	2 (3)	2 (4)	2 (3)	1
Time with friends	1 (3)	1 (3)	1 (3)	0
Mood	2 (4)	1 (4)	2 (5)	1
Physical activities	3 (5)	3 (4)	3 (5)	1
Sleep	2 (3)	1 (3)	2 (5)	2
**PROMIS-25** ^**3**^ **(*n =*** **28)**				
Mobility	40 (12)	41 (13)	27 (18)	41
Anxiety	50 (11)	49 (7)	55 (21)	44
Depressive symptoms †	52 (11)	52 (6)	59 (11)	44
Fatigue	51 (15)	54 (10)	50 (17)	41
Peer relationships †	47 (18)	48 (17)	39 (16)	61
Pain interference	50 (14)	48 (13)	57 (14)	49

^1^ Numeric Pain Ratings Scale, Self- rating from 0 to 10: 0 (no pain) and 10 (worst possible pain). The child was asked to rate the intensity when the pain was at its average, lowest, and at its highest. ^2^ Pain Interference Index, Self-rating from 0 to 6: 0 (not at all) and 6 (very high). ^3^ PROMIS-25, Self-rating from 0 to 4: 0 (never) and 4 (always). Median T-scores are presented for all children and each OI-type: I, III and IV. Interquartile range (IQR) for all children and type I and III (too few in type IV). † Two missing answers for depressive symptoms and one missing for peer relationships

The total median raw score for self-perceived health status in the total sample (*n* = 28) was 40 (IQR 27). Children with type III reported lower in the mobility domain as compared to type I and IV (*p =* 0.016). No differences regarding self-perceived health status were found between sex or age groups (table 2).

A high correlation was observed between total median in PII and total median raw score in PROMIS-25 (r_s_ = 0.84, *p <* 0.001). Separate domains within PII correlated with T-scores in separate domains within PROMIS-25 (table 3).

**Table 3:** Correlations between Pain Interference Index (PII) (*n =* 27) and Patient Reported Outcome Measurement Information System Pediatric-25 Profile v1.1 (PROMIS-25) (*n =* 28)

Variable	PROMIS-25 ^**2**^	Mobility	Anxiety	Depressive symptoms †	Fatigue	Peer relationships †	Pain interference
**PII** ^**1**^							
Schoolwork		r_s_ = −0.20 *p =* 0.310	r_s_ = 0.50 *p =* 0.003**	r_s_ = 0.49 *p =* 0.012*	r_s_ = 0.43 *p =* 0.023*	r_s_ = −0.54 *p =* 0.004**	r_s_ = 0.51 *p =* 0.006**
Leisure activities		r_s_ = −0.51 *p =* 0.006**	r_s_ = 0.43 *p =* 0.024*	r_s_ = 0.54 *p =* 0.005**	r_s_ = 0.39 *p =* 0.043*	r_s_ = −0.23 *p =* 0.270	r_s_ = 0.58 *p =* 0.001**
Time with friends		r_s_ = −0.50 *p =* 0.007**	r_s_ = 0.41 *p =* 0.031*	r_s_ = 0.51 *p =* 0.008**	r_s_ = 0.47 *p =* 0.012*	r_s_ = −0.37 *p =* 0.060	r_s_ = 0.58 *p =* 0.001**
Mood		r_s_ = −0.43 *p =* 0.025*	r_s_ = 0.77 *p <* 0.001**	r_s_ = 0.72 *p <* 0.001**	r_s_ = 0.36 *p =* 0.058	r_s_ = −0.56 *p =* 0.003**	r_s_ = 0.54 *p =* 0.003**
Physical activities		r_s_ = −0.32 *p =* 0.100	r_s_ = 0.20 *p =* 0.310	r_s_ = 0.23 *p =* 0.250	r_s_ = 0.10 *p =* 0.580	r_s_ = −0.077 *p =* 0.70	r_s_ = 0.52 *p =* 0.005**
Sleep		r_s_ = −0.38 *p =* 0.049*	r_s_ = 0.52 *p =* 0.005**	r_s_ = 0.54 *p =* 0.004**	r_s_ = 0.31 *p =* 0.110	r_s_ = −0.41 *p =* 0.038*	r_s_ = 0.60 *p =* 0.001**

Spearman’s correlation coefficient (r_s_) was calculated between PII (*n =* 27) and PROMIS-25 (*n =* 28). Significant at *p <* 0.05 (⁕ < 0.05, ⁕⁕ < 0.005) and r > 0.37. ^1^ PII consists of a self-rating from 0 to 6, where 0 means “not at all” and 6 “very high” pain interference during six activities: schoolwork, leisure activities, time with friends, mood, physical activities, and sleep. ^2^ PROMIS-25 is a self-rating instrument with the domains mobility, anxiety, depressive symptoms, fatigue, peer relationships and pain interference. The rating is on a scale from 0 to 4 where 0 means never and 4 always. † Two missing answers for depressive symptoms and one for peer relationships

## Discussion

The main finding was that pain was present in almost all children, regardless of OI-type, sex, and age group. In addition, pain interfered in children’s everyday life, which was associated with children’s self-perceived health status.

Almost all children reported presence of pain and the intensity was consistent with previous studies including children with OI [23, 24]. In the present group, more than 60% received treatment with intravenous bisphosphonates, which also have a possible analgesic effect, indicating an even higher pain intensity unless the treatment was not carried out. However, many children with OI have experienced pain from an early age and might have adjusted their life by avoiding activities in order to reduce pain or have adapted to a certain level of chronic pain, and therefore some children might have underestimated their level of pain [16]. Children reported presence of pain several times per week. The findings are in line with results from the UK, where a random sample of 35 children with OI, aged 5–18 years was included [11]. Concerning pain location, the present study showed that the back and feet were most common. Zack et al. also described the back as the most common location, together with the chin and front of the thighs [11]. In the present study children reported the probable causes of pain, of which the most frequent were walking long distances, followed by walking, exercise, football, and jumping. Children and youth were rather specific when they described the “usual pain” (chronic pain) and if there was some new suddenly arising and unfamiliar pain (acute pain) they could clearly describe the new pain and the incident that caused the new pain (acute pain). However, many children further explained that prolonged sedentary positions caused pain. Confirming results were reported by Zack and colleagues, where 43% of the participants described minor traumas, recent exercise, or prolonged poor positions as painful situations [11].

The total mean PII score was 11 in the group of children with OI (mean age: 11 years), in comparison to a mean PII score of 8.7 in children with cerebral palsy (mean age: 7.3 years), which was reported in a population-based study [25]. In addition, in a group of adolescents with long-standing pain (mean age: 14.1 years) the mean PII score was 18, and the authors discovered higher PII scores in older participants [20]. These results indicate a risk for increased pain over time. In the present study, pain interfered in children’s everyday lives, and affected participation in various activities. Since physical activity is a key factor for decreasing bone resorption and rebuilding bone mass, this information is essential. If children avoid physical activities because of fear of pain, or due to pain, it might cause a vicious circle of inactivity, which further decreases bone density and increase the risk of fractures, additional pain, and further inactivity [14, 26].

Many children reported pain interference during sleep, which has been described earlier in an integrative review concerning pain experiences in children and adolescents with OI [13]. Zack et al. reported that the impact of pain was most frequent in participants when they were trying to fall sleep [11]. Altogether, these results indicate that sleep is a topic that should be further investigated, since sleep deprivation affects school performance, emotional status, relationships, and by increase the pain experience itself [10, 25, 27].

In our sample, pain interference during schoolwork was associated with anxiety, depressive symptoms, fatigue, and less social support from friends. Confirmatory results were described from the UK [11]. The frequency of children reporting school absence due to pain was high in our group of children. Many previous studies have used “school absence”, to evaluate children’s ability to participate in school-related activities, since they are considered to be the most important activities for children, due to social, cognitive, emotional, and physical aspects [28].

Self-perceived health status was investigated, and mobility was reduced in the total sample, and the lowest mobility was detected in children with type III, while a positive trend was that many children reported results similar to the general population in other domains, findings consistent with previous research [29, 30]. Confirming results were also presented in a review concerning QoL in children and adults with OI, where the authors concluded that physical QoL appeared to be lower than in the general population, while the mental and psychosocial QoL was equal or better [31]. Both low physical and social scores in HRQoL were detected in a Brazilian prospective cross-sectional study, including 52 children and adolescents with OI, aged 5–17 years; however, significant differences were detected between the OI types [29]. A lower HRQoL in children with OI than children in the general population, and especially those with severe OI-types, was reported in a Chinese cross-sectional study, including 138 children with OI, aged 2–18 years [30]. No difference between sexes was found in the present study, which could imply that the self-perceived health status might be similar for boys and girls within the Swedish OI population, a finding consistent with the Chinese study [30]. In our group of children, a high correlation was detected between children’s self-perceived health status and pain interference in everyday life, a discovery emphasising the importance of finding optimal possibilities for treatments. The results are in line with previous research [29].,

A limitation in this study was the small sample included, and a reason is that at our clinic a high number of children are below the age of 6 years, and in addition small sample is to be expected in single-site studies of children with rare diseases [31]. A small sample risks the external validity, and reduce the possibility to generalize the results, however, a strength in the present study was that the representation of OI-types was consistent with the prevalence in the Swedish population [1]. In addition, the findings concerning presence of pain were mainly consistent with previous studies. Another limitation was the use of the five structured questions in the interview, that had not previously undergone psychometric testing. Strengths were that only one child declined to participate, that the response rate in the questionnaires was high, and that the same researcher (KL) conducted all interviews. The use of PII, a psychometric tested index, considered as an adequate tool to assess pain interference in children and adolescents, was a further strength [20]. Another strength was the use of PROMIS-25 since it offers possibilities for comparison with the general population.

### Future studies

It is desirable to investigate interventions that reduce pain in children with OI in future studies. Pain interference and sleep is another topic that could be further assessed for the OI-population.

## Conclusions

In conclusion, almost all children experienced pain, which interfered in children’s everyday lives, affected participation in various activities and was associated with reduced self-perceived health status. If children avoid physical activities because of pain, it might cause a vicious circle of inactivity, which further decreases bone density and increase the risk of fractures. The results emphasize the importance to offer adequate pain reducing interventions.

## Data Availability

The dataset analysed during the current study are not publicly available due to ethical concerns but are available from the corresponding author on reasonable request.

## Abbreviations

HRQoL: Health-related quality of life
NRS: Numerical Rating Scale
OI: Osteogenesis Imperfecta
PII: Pain Interference Index
PROMIS-25: Patient Reported Outcome Measurement Information System Pediatric-25 Profile v1.1
QoL: Quality of Life
